# Evaluating ChatGPT-4’s historical accuracy: a case study on the origins of SWOT analysis

**DOI:** 10.3389/frai.2024.1402047

**Published:** 2024-05-03

**Authors:** Richard W. Puyt, Dag Øivind Madsen

**Affiliations:** ^1^Industrial Engineering and Business Information Systems (IEBIS), Faculty of Behavioural, Management and Social Sciences (BMS), University of Twente, Enschede, Netherlands; ^2^Department of Business, Marketing and Law, USN School of Business, University of South-Eastern Norway, Hønefoss, Norway

**Keywords:** ChatGPT, SWOT analysis, historical analysis of management concepts, AI in management research, strategy tools

## Abstract

In this study we test ChatGPT-4’s ability to provide accurate information about the origins and evolution of SWOT analysis, perhaps the most widely used strategy tool in practice worldwide. ChatGPT-4 is tested for historical accuracy and hallucinations. The API is prompted using a Python script with a series of structured questions from an Excel file and the results are recorded in another Excel file and rated on a binary scale. Our findings present a nuanced view of ChatGPT-4’s capabilities. We observe that while ChatGPT-4 demonstrates a high level of proficiency in describing and outlining the general concept of SWOT analysis, there are notable discrepancies when it comes to detailing its origins and evolution. These inaccuracies range from minor factual errors to more serious hallucinations that deviate from evidence in scholarly publications. However, we also find that ChatGPT-4 comes up with spontaneous historically accurate facts. Our interpretation of the result is that ChatGPT is largely trained on easily available websites and to a very limited extent has been trained on scholarly publications on SWOT analysis, especially when these are behind a paywall. We conclude with four propositions for future research.

## 1 Introduction

The rising interest in ChatGPT from both the public and the media has been noteworthy, signaling a burgeoning field of research that has seen a near-exponential surge (Farhat et al., 2024), particularly within the realms of business and management (Rane, 2023; Elbanna and Armstrong, 2024). Numerous studies have explored the potential applications of ChatGPT-4, aiming to utilize its capabilities to enhance the efficiency of diverse business processes. Examples include explorations into ChatGPT-4’s utility in streamlining customer service, decision-making, higher education, marketing and operations (Dwivedi et al., 2023; Loos et al., 2023), as well as its competency in tackling examination questions across various business disciplines (Terwiesch, 2023; Wood et al., 2023).

However, there exists a notable gap in the literature: the scarcity of critical and reflective examinations of ChatGPT-4’s outputs from a management history perspective. Given the significance of understanding the origins and evolution of management thought (Wren and Bedeian, 2023), an analysis of how Artificial Intelligence (AI) chatbots like ChatGPT-4 interpret and respond to inquiries about fundamental management concepts and ideas could provide valuable insights. At the outset, we had a hunch that ChatGPT-4 could potentially struggle with recounting the origins and history of SWOT analysis since its history is murky, even in the peer-reviewed academic literature (Helms and Nixon, 2010; Benzaghta et al., 2021). Research has found that there is considerable confusion and misunderstanding of SWOT’s origins even among scholars publishing on the topic (Madsen, 2016; Puyt et al., 2023).

It is important to understand the ability of AI models like ChatGPT to retrieve valid knowledge, for example about management history. This is especially the case in the age of ChatGPT and similar chatbots since many students and practitioners turn to these technological tools and apps for answers and guidance about tools like SWOT analysis. An exploration of ChatGPT-4’s ability to provide valid answers on advanced and less clear-cut topics could provide useful insight that could be of use to those developing Large Language Models (LLM). Studies like ours could play an important role in informing the ongoing development and refinement of AI chatbots, ensuring they align more closely with the nuanced trajectories of management theories and practices. Our study also has implications for management history literature and management research more broadly, as it enriches our comprehension of AI’s potential impact on the field and the benefits and limitations of using these technologies in management education and scholarship.

In our study, we assessed ChatGPT-4’s ability to provide accurate information about the origins and evolution of SWOT analysis. SWOT analysis is arguably a very good case since studies have shown that it is a very popular tool in business practice and research (Qehaja and Kutllovci, 2020; King et al., 2023). Therefore, it is of great interest to find out the extent to which ChatGPT-4 is providing accurate information about the history and origins of SWOT analysis. Specifically, we compared the responses of ChatGPT-4 to a series of assertions in the literature that we know are inaccurate (but pervasive). For example: 1. The origins of SWOT analysis can be traced back to the Harvard Business School in the 1960s, where it emerged from discussions in the classroom (Chermack and Kasshanna, 2007; Leih and de los Reyes, 2023), 2. SWOT analysis was initially introduced in 1965 in the book *Business Policy: Text and Cases* (Learned et al., 1965), a case book written by Harvard Business School Professors in the 1960s (Barney, 1995; Jarzabkowski and Kaplan, 2015) and 3. SWOT analysis is developed in the 1960s by Albert S. Humphrey (Jain, 2015; Ojala, 2017; Teoli and An, 2019; Myllylä and Kaivo-oja, 2024).

To conduct an evaluation, we developed a Python script to systematically prompt ChatGPT-4 API, using a series of questions, designed to probe its knowledge base and assess its proficiency in recounting the historical background and conceptual contributions to SWOT analysis. We manipulated the settings of the two main parameters that control the creativity of the responses in ChatGPT-4 (Temperate and Top_p) to find out to what extent they affect the differences in the quality of the responses to our prompts. The responses were recorded in an Excel spreadsheet and the accuracy of the responses was rated.

The rest of the paper is structured in the following way. In Section 2 we provide a brief overview of SWOT Analysis’ origins and significance. In Section 3 we describe the methodology. Section 4 presents the results. In Section 5 we discuss the results considering existing literature. Finally, in Section 6 we conclude the paper and suggest propositions for follow-up studies.

## 2 SWOT analysis: origins and significance

In this section, we provide an overview of the origins and significance of SWOT analysis. We start by briefly explaining the basics. SWOT is an acronym for Strengths, Weaknesses, Opportunities and Threats (Hansler, 1988). The present-day SWOT analysis is depicted as a 2 × 2 matrix used for brainstorming (Piercy and Giles, 1989; Valentin, 2001). It is widely used in businesses and organizations to map out their strengths (what they excel at), weaknesses (areas needing improvement), opportunities (chances in the environment to grow or excel), and threats (external challenges to overcome). Its application can be found in business strategy (Pickton and Wright, 1998), marketing planning (Fahy and Smithee, 1999), change management (Heusinkveld and Benders, 2012) as well as personal development (Bailey, 1999). SWOT analysis can be called the evergreen strategy tool for environmental scanning. Its popularity is evident through mainstream appeal, exemplified by the tool’s feature on the TV show Silicon Valley. Moreover, another characteristic of SWOT is its simplicity and the fact that it has been transformed into a verb (‘Let us SWOT it’).

The original SWOT analysis, denoted as the SOFT approach (Stewart et al., 1965), along with its modern rendition featuring a 2 × 2 matrix in a cruciform shape (Argenti, 1974) are both notably underrepresented in the literature. Variations like Threat/Opportunity analysis (Ansoff, 1975), WOTS-UP analysis (Steiner and Miner, 1977) and the TOWS matrix (Weihrich, 1982) are widely recognized in the literature; however, they lack theoretical linkage to the original SOFT approach.

In the 1980s, a paradigm shift from business policy to strategic management led to the adaptation of economists’ language and models (Bower, 1982). SWOT analysis went out of fashion in the scholarly literature and has undergone several significant changes since its introduction. As a consequence, a disconnect in theory development within the strategic management literature has emerged (Hofer and Schendel, 1978; Hambrick and Chen, 2008). Since the 1990’s, the dominant logic in the field of strategic management has been the resource-based view of the firm, a perspective inspired by economic theory (Wernerfelt, 1984, 1995; Barney, 1991). This perspective is rooted in the LCAG framework (Christensen et al., 1978; Andrews, 1980). Through revisionism and reification, the LCAG framework or Andrew’s strategy framework (De Los Reyes, 2011; Bell and Rochford, 2016) is often misattributed as the origins of SWOT analysis (Mintzberg, 1990; Barney, 1995; Argyres and McGahan, 2002; Bower, 2008). Due to this, there exists scant consensus and documentation regarding the tool’s history and development (Bell and Rochford, 2016).

During the turn of the millennium, notable innovations in SWOT analysis emerged from other models and techniques. This period marked the rise of hybrid SWOT models such as A’WOT (Kurttila et al., 2000), Dual-perspective SWOT (Novicevic et al., 2004) and Meta-SWOT (Agarwal et al., 2012).

Over the past 30 years, but probably longer, SWOT analysis has been reported as the number one strategy tool in practice (Webster et al., 1989; Rigby, 2001; Stenfors et al., 2007; Bellamy et al., 2019). Despite its popularity and widespread use among practitioners, strategy scholars criticize SWOT analysis for being a traditional or even a simplistic classificatory device (Haberberg, 2000; Arnaud et al., 2016; David et al., 2021) and even repeat the assertion that it has ‘little intellectual content’ (Jarzabkowski and Kaplan, 2015, p. 542).

In our view, the enormous popularity and widespread diffusion of SWOT in business practice and business school education (Farrokhnia et al., 2023), as well as the relatively broad and mainstream appeal of the tool, makes it all the much more important to ensure that the history and origins are correctly understood and recounted. This is especially the case in the age of ChatGPT and cognoscenti (Borges, 2023), since many students and practitioners turn to this technology for answers and guidance about tools like SWOT.

## 3 Methods

We prepared for the evaluation by manipulating two key parameters, Temperature and Top_p, which control the level of creativity or randomness in the responses generated by the ChatGPT-4 API from OpenAI.com. The Temperature setting ranges from 0.0 to 2.0, while the Top_p setting ranges from 0.0 to 1.0. Notably, only one variable can be adjusted at a time when prompting the API with a Python script, necessitating a comparison between default settings (Temperature 1.0; Top_p 0.5) and stricter settings (Temperature 0.2; Top_p 0.1). Our aim was to assess the response quality across different settings, with an initial hypothesis that stricter settings would produce more reliable outcomes. However, at the outset, there was uncertainty related to determining which variable setting yields the most trustworthy results.

To carry out the evaluation, we followed a systematic approach to data gathering and analysis. Initially, we selected 50 relevant publications on SWOT analysis spanning from 1965 to 2019. Subsequently, we extracted all pertinent data necessary for grading ChatGPT 4 responses - such as Author(s), Outlet, Pages, Title, and Year, and compiled them into a reference list. We formulated standard questions based on the first 50 authors and developed and tested a Python script with appropriate parameters. We then prompted the API using these standard questions loaded from an Excel file. The resulting responses were recorded in a separate Excel file. We then graded these responses on a binary scale for both default and strict settings, with particular attention paid to identifying hallucinations and traces of training data sources. Further analysis included investigating the availability of scholarly publications online, constructing descriptive statistical analyses with normal distributions, generating descriptive tables, and finally, formulating propositions for future research.

## 4 Results

### 4.1 Response quality vs. Scopus citations

Our assessment of ChatGPT-4’s responses is based on a rating of five elements (Author, Outlet, Pages, Title and Year) on a binary scale (0–1). The overall response quality is found to be quite unsatisfactory and does not correlate with evidence found in the top 5 most cited papers in our sample, based on the volume of citations found in the Scopus database (Table 1). This discrepancy highlights a significant gap between the quantity of referenced material in a reputable academic database and the quality of the generated responses. It suggests that despite the potential wealth of information available in Scopus citations, this depth and breadth of knowledge are not effectively reflected in the quality of the responses produced, indicating a potential area for improvement in source integration.

**Table 1:** Top 5 most popular SWOT variations in the literature (1980–2020).

**Nr.**	**Variation**	**Year**	**Scopus**	**Source / Publisher**
1	VRIO framework	1991	25,821	Journal of management
2	ANP-SWOT	2007	404	Information sciences
3	Threat/Opportunity analysis	1975	395	California management review
4	TOWS matrix	1982	381	Long range planning
5	SMART SWOT	2017	177	Int. Journal of Cont. Hosp. Mgt.

We further explored whether the top 5 most popular SWOT variations in our sample (1980–2020) are available via open access or whether they are behind a paywall. All these articles are published in traditional subscription-based journals. However, in the cases of some of the articles, it is easy to find as a PDF via Google Scholar, and in one case the article is posted in full-text on ResearchGate (see Table 2).

**Table 2:** Availability of the top 5 most popular SWOT variations in the literature (1980–2020).

**Nr.**	**Variation**	**ResearchGate**	**Pdf via Google**	**Access to paper**
1	VRIO framework	Request	Yes	Subscription-based
2	ANP-SWOT	Request	Yes	Subscription-based
3	Threat/opportunity analysis	No	No	Subscription-based
4	TOWS matrix	No	Yes	Subscription-based
5	SMART SWOT	Yes	No	Subscription-based

Looking ahead to future investigations, we put forward the following proposal:

Proposition 1: *Improving the incorporation of Scopus citations into the training dataset is anticipated to beneficially impact the response quality. This effect will be evaluated using a binary scale (0–1) across five dimensions: Author, Outlet, Pages, Title and Year.*

### 4.2 Response quality vs. variable settings

**Figure 1:**
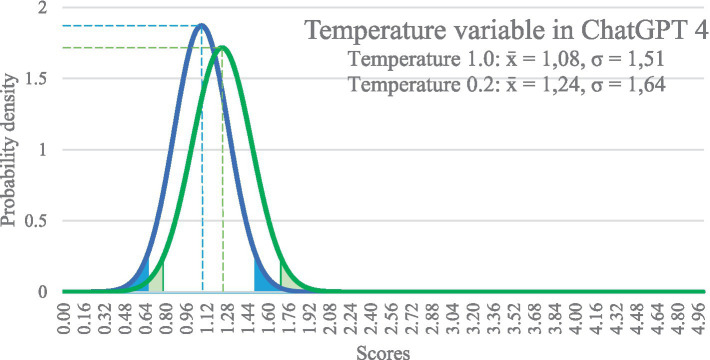
Quality of the responses to prompts after adjusting the temperature variable.

The Temperature variable of ChatGPT is normally set to 1.0 (0.0–2.0) and strikes a balance between the accuracy and creativity of the generated responses. However, the quality of the responses did not significantly improve when we changed the Temperature variable to a stricter setting of 0.2 (see Figure 1). We expected to see a much higher level of accuracy in the responses.

**Figure 2:**
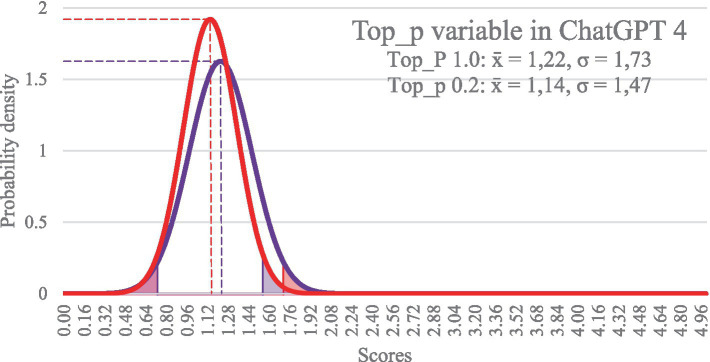
Quality of the responses to prompts after adjusting the Top_p variable.

We compared our approach by testing the responses against the Top_p (or nucleus sampling) variable. The Top_p setting is normally set to 0.5 (0.0–1.0) and helps to control the word (AiPromptsKit, 2023). Here, we also expected to see an increase in accuracy when we changed the Top_p variable to 0.2. However, the quality of the responses even deteriorated slightly (see Figure 2). The different settings of the parameters Temperature and Top_p initially yielded very little differences in the quality of the responses to the prompts.

Upon close inspection of all the responses, we discovered spontaneous accurate historical facts about the origins of SWOT analysis. Initially, there was no pattern to be discerned even when we changed the settings of the parameters. However, ChatGPT-4 sometimes leaked fragments of the sources of its training data. For instance (partial) URLs (e.g., https://rapidbi.com/swotanalysis/ or https://www.businessnews), but also examples of hallucinations (e.g., ‘Albert Humphrey (1926–2005).” In Key Thinkers in Critical Management Studies, edited by Martin Parker, Robyn Thomas, and Martyna Śliwa, 147–152. London: SAGE Publications Ltd., 2016. doi: 10.4135/9781473957953.n19’ or ‘Weihrich, H (2005). Swot analysis. Encyclopedia of Management)’. Many instances of hallucinations in other fields have been reported in the ChatGPT literature (Alkaissi and McFarlane, 2023; Metze et al., 2024; Siontis et al., 2024).

Moving forward, we suggest the following hypothesis for further study:

Proposition 2: *Modifying the Temperature and Top_p parameters to stricter settings in a language model markedly enhances the response quality to prompts.*

Table 3 shows spontaneous historically accurate facts about the historical background of SWOT. As can be seen, regardless of the Temperate and Top_p parameters, ChatGPT-4 is not able to reference the SOFT approach (No. 1). The same is the case for No. 5, which only shows up 2% of the time under TP 0.5. Other facts related to Long Range Planning Service subscribers only show up between 2 and 8% of the time. When it comes to No. 3, Stanford Research Institute, ChatGPT-4 references this 12–16% of the time. Finally, Albert S. Humphrey is referenced between 22–30% of the time. Table 3 illustrates that the variances among the various configurations are comparatively minor. Overall, it is unclear where these references come from.

**Table 3:** Spontaneous historical accuracy in the responses to different variable settings.

**No.**	**References found in the data**	**T 1.0**	**T 0.2**	**TP 0.5**	**TP 0.1**
1	Robert F. Stewart	0%	0%	0%	0%
2	Albert S. Humphrey	30%	24%	22%	24%
3	Stanford Research Institute	16%	16%	16%	12%
4	Long range planning service	2%	4%	8%	6%
5	SOFT approach (the original SWOT analysis)	0%	0%	2%	0%

Table 4 provides an overview of the best-scoring SWOT variations for different settings of the Temperature and Top_p parameters. We can see a strange pattern of scores based on the different settings of these parameters. There are several possible explanations for these results, such as the age of the publication (range: 1975 to 2019), the number of citations, or the accessibility of the paper. However, based on our judgment, it seems rather random and there is no clear pattern.

**Table 4:** Best scoring SWOT variations according to different variable settings (5/5 points).

**Nr.**	**Variation**	**Year**	**T 1.0**	**T 0.2**	**TP 0.5**	**TP 0.1**
1	VRIO framework	1991	X	X	X	X
2	ANP-SWOT	2007	X	X	X	X
3	Threat/Opportunity analysis	1975	X	X	X	X
4	TOWS matrix	1982	X	X		
5	SMART SWOT	2017		X	X	
6	SWOPT	1993			X	
7	Importance/Performance Analysis	2019			X	

After close inspection of the responses, the sources of ChatGPT-4’s training data about the history of SWOT analysis can be triangulated to three sources on the Internet: Businessballs Ltd., Marketingteacher Ltd. and Rapidbi Ltd (See Table 5). On businessballs.com, there are elements of historically accurate information about the origins of SWOT, and there are discussion of the role of Albert S. Humphrey in the development of SWOT. The website marketingteacher.com has a list with 9 references to SWOT-related articles, including a ghost reference called: *Humphrey, A.S (1960). SWOT for Management Consulting, SRI Consulting Business Intelligence*. This document does not exist and cannot be found in the archives of SRI Consulting Business Intelligence. We can also find a full reference to the TOWS matrix. Finally, rapidbi.com has interesting contributions about myths in the history of SWOT analysis, demonstrating the value of studying source material.

**Table 5:** Potential training sources for ChatGPT-4 regarding the history of SWOT analysis.

**Nr.**	**SWOT training sources**	**Title and authors**
1	Businessballs Ltd.	SWOT Analysis (Chapman, 2011)
2	Marketingteacher Ltd.	SWOT analysis (Friesner, 2011)
3	Rapidbi Ltd.	History of SWOT analysis (Morrison, 2015, 2012)

Future research could investigate the impact of diversifying the range of sources in AI training data on the quality and breadth of knowledge related to business analysis tools such as SWOT analysis. Specifically, this research could hypothesize that incorporating a wider array of authoritative sources beyond ideas from consultancies like Businessballs Ltd., Marketingteacher Ltd., and Rapidbi Ltd. will enhance ChatGPT-4’s generative capabilities and presentation of SWOT analysis, potentially leading to a more nuanced and comprehensive insights into the history and evolution of management concepts and ideas.

Moving forward, we suggest the following hypothesis for further study:

Proposition 3: *Incorporating a broader spectrum of authoritative sources into the training data of AI models like ChatGPT-4 will significantly improve their accuracy and depth of insights when generating content on SWOT analysis.*

### 4.3 Lack of scholarly training data

ChatGPT-4’s training data does not include scholarly publications on SWOT analysis, indicating a lack of direct access to academic databases and journals, especially to articles that are behind a paywall and where no full-text is readily available via Google or a preprint server. This limitation suggests that while the model can provide information on SWOT analysis, its responses might not reflect the latest research or incorporate in-depth analyses found in scholarly work. As a result, the depth and currency of ChatGPT 4’s knowledge of SWOT analysis could be primarily shaped by more generally accessible sources on the internet, rather than peer-reviewed academic studies. It seems that academic documents, such as PDFs or Word files, detailing the history or development of SWOT analysis, were not included in the training data for ChatGPT-4.

Moving forward, we suggest the following hypothesis for further study:

Proposition 4: *Granting ChatGPT 4 access to scholarly databases and including academic publications in its training data will enhance its accuracy and depth of knowledge on SWOT analysis.*

## 5 Discussion

Our findings present a nuanced view of ChatGPT’s capabilities in the field of management history. We observe that while ChatGPT-4 demonstrates a high level of proficiency in describing and explaining the general concept of SWOT analysis and in terms of carrying out relatively straight-forward analyses using the framework, it performs worse on advanced tasks, such as detailing the concept’s history and origins. We found that there are notable discrepancies when it comes to detailing the origins of SWOT analysis. These inaccuracies range from minor factual errors to more significant hallucinations (Ahmad et al., 2023; Giuffrè et al., 2023; Moritz, 2024), where the AI fabricates elements of the strategy tool’s history. In addition, there might be another explanation for the accuracy of some results. Apart from harvesting training data from a few topical websites, it is also possible that references to scholarly articles are harvested from Google Scholar (scholar.google.com). Scholar Google indexes pre printer servers like ArXiv, SSRN or SocArXiv and provides direct links to PDF files.

Our research allows us to situate these findings within the expansive realm of Large Language Models (LLMs), with a specific emphasis on ChatGPT. During our investigation, we noted that ChatGPT’s responses to prompts on the conceptual contributions on SWOT analysis were somewhat lacking in accuracy. This inaccuracy could be attributed, in part, to the inherently complex and obscure history of SWOT Analysis, where there is little consensus even among experts in the area. Although exploring these inaccuracies across other management theories was beyond the purview of our study, it is plausible that similar discrepancies could be identified in ChatGPT’s treatment of other management concepts and ideas, such as PEST analysis, stakeholder analysis or scenario planning. For training the generative training data, access to open-access publications could be a good start. Supervised finetuning could be done by panels of topical experts, similar to a peer review process.

## 6 Conclusion

In conclusion, this study contributes to the ongoing debate about the reliability and usefulness of AI language models in academic and professional settings. Our findings not only add to our understanding of ChatGPT’s capabilities and limitations but also paves the way for future research on improving AI’s role in supporting strategic management education and practice.

The implications of our study are three-fold. Firstly, it highlights the potential of AI language models like ChatGPT as valuable resources for educational and professional purposes, offering instant access to a wealth of knowledge. It is, however, key that the information provided by the chatbots is accurate and that hallucinations are minimized. If not, the chatbots will perpetuate falsehood and contribute to the longevity of academic urban legends (McGee, 1985; Rekdal, 2014). Secondly, it underscores the critical need for users to approach AI-generated information cautiously, especially when dealing with complex historical data where accuracy is paramount.

Thirdly, our findings underscore the necessity for expert involvement in the training of AI models to enhance their proficiency in specialized fields such as business and management. This opens the door to the potential creation of chatbots specifically designed for business and management applications, offering tailored insights and analyses.

### 6.1 Limitations and future work

Given the exploratory nature of our investigation, it is important to acknowledge certain limitations and outline potential directions for future research. One limitation is that there is a risk of bias in our evaluation of ChatGPT’s responses. Ideally, we would like the multiple independent reviewers to rate the answers provided by ChatGPT-4. However, given our exploratory aims, we faced some constraints and had to make trade-offs. The risk of bias is also mitigated by the fact that we have expert-level knowledge of the topic area.

One significant area for further exploration is the comparative analysis of different AI chatbots, such as ChatGPT versus Google Gemini, particularly in their handling of management history. Such a study would aim to discern whether these AI chatbots show convergence or divergence in their responses and how they describe management concepts.

Another intriguing line of inquiry involves the comparison of ChatGPT’s proficiency in terms of describing various management and strategic planning tools against other AI chatbots. Specifically, an examination of how these AI models recount the origins and evolution of strategy tools like PEST analysis (Johnson and Scholes, 1995), could yield interesting insights. Like SWOT analysis, this environmental scanning tool also stems from the dawn of long-range planning. It is speculated that Francis J. Aguilar was the first to mention the factors of a strategic environmental analysis: Economic, Technological, Social and Political, which became known by the acronym ETSP (Aguilar, 1965).

Finally, our findings call for further studies to explore the mechanisms behind the inaccuracies and hallucinations observed in AI responses, aiming to enhance the fidelity of information provided by such technologies.

## Data availability statement

The data presented in the study can be found in the Supplementary material.

## Author contributions

RP: Conceptualization, Data curation, Formal Analysis, Investigation, Methodology, Software, Visualization, Writing – original draft, Writing – review & editing. DM: Conceptualization, Validation, Writing – original draft, Writing – review & editing.
